# Application of environmental DNA to detect an endangered marine skate species in the wild

**DOI:** 10.1371/journal.pone.0178124

**Published:** 2017-06-07

**Authors:** Kay Weltz, Jeremy M. Lyle, Jennifer Ovenden, Jessica A. T. Morgan, David A. Moreno, Jayson M. Semmens

**Affiliations:** 1 Fisheries and Aquaculture Centre, Institute for Marine and Antarctic Studies, University of Tasmania, Hobart, Tasmania, Australia; 2 Molecular Fisheries Laboratory, School of Biomedical Sciences, University of Queensland, Brisbane, Queensland, Australia; 3 Queensland Alliance for Agriculture and Food Innovation, University of Queensland, Brisbane, Queensland, Australia; University of Hyogo, JAPAN

## Abstract

Environmental DNA (eDNA) techniques have only recently been applied in the marine environment to detect the presence of marine species. Species-specific primers and probes were designed to detect the eDNA of the endangered Maugean skate (*Zearaja maugeana*) from as little as 1 L of water collected at depth (10–15 m) in Macquarie Harbour (MH), Tasmania. The identity of the eDNA was confirmed as *Z*. *maugeana* by sequencing the qPCR products and aligning these with the target sequence for a 100% match. This result has validated the use of this eDNA technique for detecting a rare species, *Z*. *maugeana*, in the wild. Being able to investigate the presence, and possibly the abundance, of *Z*. *maugeana* in MH and Bathurst harbour (BH), would be addressing a conservation imperative for the endangered *Z*. *maugeana*. For future application of this technique in the field, the rate of decay was determined for *Z*. *maugeana* eDNA under ambient dissolved oxygen (DO) levels (55% saturation) and lower DO (20% saturation) levels, revealing that the eDNA can be detected for 4 and 16 hours respectively, after which eDNA concentration drops below the detection threshold of the assay. With the rate of decay being influenced by starting eDNA concentrations, it is recommended that samples be filtered as soon as possible after collection to minimize further loss of eDNA prior to and during sample processing.

## Introduction

Determining the presence of endangered marine species within an area that is heavily impacted by anthropogenic processes (e.g. fish farming or commercial and recreational fishing), is important for the implementation of effective management strategies to minimize such impacts on the particular population [1]. Confirming presence relies on locating the animals, which can prove challenging for species with low population numbers [2]. A variety of methods have been used to determine the presence of rare marine species, including fishing and underwater visual surveys [3]. Genetics has proven an alternate technique for detecting the presence of rare or cryptic species in the wild, by using DNA occurring in environmental samples of sediments, ice or water [4–9]. Environmental DNA (eDNA) has been used for over a decade to investigate the presence of a variety of organisms, including microbes, plants and animals, providing information on past and present biodiversity [9]. Vertebrate eDNA is DNA that is deposited in the environment through a variety of bodily processes, including the shedding of skin and/or hair and/or feathers, or excretion of defecation and/or urination and/or saliva [4,9].

In the aquatic environment, the presence of a rare species in an area is assessed by taking a water sample and testing whether the DNA of the target species is present in the sample [4]. Using eDNA to determine presence of an aquatic species has potential advantages over alternate approaches as there is no need to physically locate and/or capture individuals to determine whether it is present in the area [10–14]. Additionally, developing a species-specific eDNA assay requires only a single DNA sample of the target species from which genetic primers are designed. A disadvantage to using eDNA is that it is generally present in very low concentrations and decays over time in the environment due to UV radiation and hydrolysis by bacteria during DNase activity [15].

When using eDNA to determine species presence in aquatic environments, it is important to consider not only the rate at which eDNA decays *in situ* but also in the water samples once collected. Along with the influence of biomass and proximity of target species, the *in situ* decay of eDNA will determine the quantity of eDNA available in the collected water sample. Once collected, further decay will influence how long, between obtaining and processing the sample, the target eDNA can persist before concentrations drop to below the detection limit of the assay.

Since the initial application of eDNA in aquatic environment to determine microbial biomass, the technique has been developed into multi-species and species-specific approaches, which have been used to target larger aquatic organisms. The multi-species approach seeks to target and amplify all of the eDNA present using Next Generation Sequencing (NGS) techniques, whereas the species-specific approach uses real-time, or quantitative, PCR (qPCR) to target a individual eDNA sequences of the target species and is confirmed through Sanger sequencing. Both approaches have been applied to detect the presence of freshwater fish and other aquatic species in pools and streams [16–19]. In recent years, eDNA techniques have also been applied in the marine environment to detect marine mammals and teleosts in the wild [4,20].

As a group, elasmobranchs (sharks and rays) are considered particularly vulnerable to overexploitation, and in recents years eDNA has been indentified as a potentially useful technique for determing species presence in the wild without having to locate or capture individuals [21–23]. To date, eDNA has been used successfully in two elasmobranch studies, to detect the endangered largetooth sawfish (*Pristis pristis*) in freshwater habitats in the Northern Territory, Australia [24] and to investigate the population genetics of the whale shark (*Rhincodon typus*) in the Arabian Gulf [8]. These studies have confirmed that eDNA can be used to determine the distribution range of the target species, and inform on population genetics [8,24].

This study further investigates the utility of eDNA as a technique to determine the presence of rare and endangered elasmobranch species by trialing this technique on the endangered Maugean skate (*Zearaja maugeana*). *Zearaja maugeana* has been classified as endangered by the IUCN and the Australian Environment Protection and Biodiversity Conservation Act (1999) based on its small population size and restricted distribution, being only reported from two remote estuarine systems off western Tasmania, Australia, namely Bathurst Harbour (BH) in the southwest coast and Macquarie Harbour (MH) in the west coast [25]. Initially discovered in Bathurst Harbour in 1988, *Z*. *maugeana* has not been recorded in that locality since 1992, despite extensive fishing and underwater visual surveys conducted over a number of years [26]. The lack of confirmed sightings has raised considerable uncertainty as to the current status of the Bathurst Harbour population, implying either a very small population size or even localised extinction.

Significantly, Bathurst Harbour has been a marine protected area since 2005 and, being in a wilderness area, is subject to minimal anthropogenic disturbance apart from small-scale mining and timber operations that occurred in the area during the early 1900s. In contrast, *Z*. *maugeana* are relatively abundant in Macquarie Harbour with a population size estimated to be around ~3000 individuals [27]. Recent studies examining the biology and habitat use of *Z*. *maugeana* in MH, have demonstrated that the main threats to the species include increased nutrient load and reduced dissolved oxygen (DO) levels, especially in the bottom waters, linked to expanding fish-farm activity within the estuary, and the incidental capture in fishing gear [27,28].

The aims of the present study were to design species-specific genetic markers, develop an eDNA assay for *Z*. *maugeana* and test the efficiency of the assay to detect eDNA of the species in the wild. For future application, the decay rate of the *Z*. *maugeana* eDNA during sample processing was also determined. Furthermore, with DO levels showing a decrease in MH, this study also investigated the affect of low DO on the degradation rate of eDNA in that water samples [27,29–31]. The benefits of being able to use this eDNA technique would include monitoring the distribution of *Z*. *maugeana* within MH as well as investigating whether a population of *Z*. *maugeana* is still present in BH. This latter consideration is especially important since traditional survey methods have failed to detect the presence of *Z*. *maugeana* at that locality for more than two decades, and being a marine protected area, the application of non-destructive sampling methods such as eDNA are especially appropriate.

## Materials & methods

All sampling was carried out in accordance with the Australian Code for the Care and Use of Animals for Scientific Purposes± 8th Edition 2013. The protocol was approved by the University of Tasmania Animal Ethics Committee (A13468) and permits 13125 and 14139 issued by the Tasmanian Department of Primary Industries, Parks, Water and Environment under Section 14 of the Living Marine Resources Management Act 1995, and permits TFA 13982, 14019 and 14253 issued under Regulation 4 of the Threatened Species Protection Regulations 2006 and Section 29 of the Nature Conservation Act 2002.

### Sample collection

**Fig 1:**
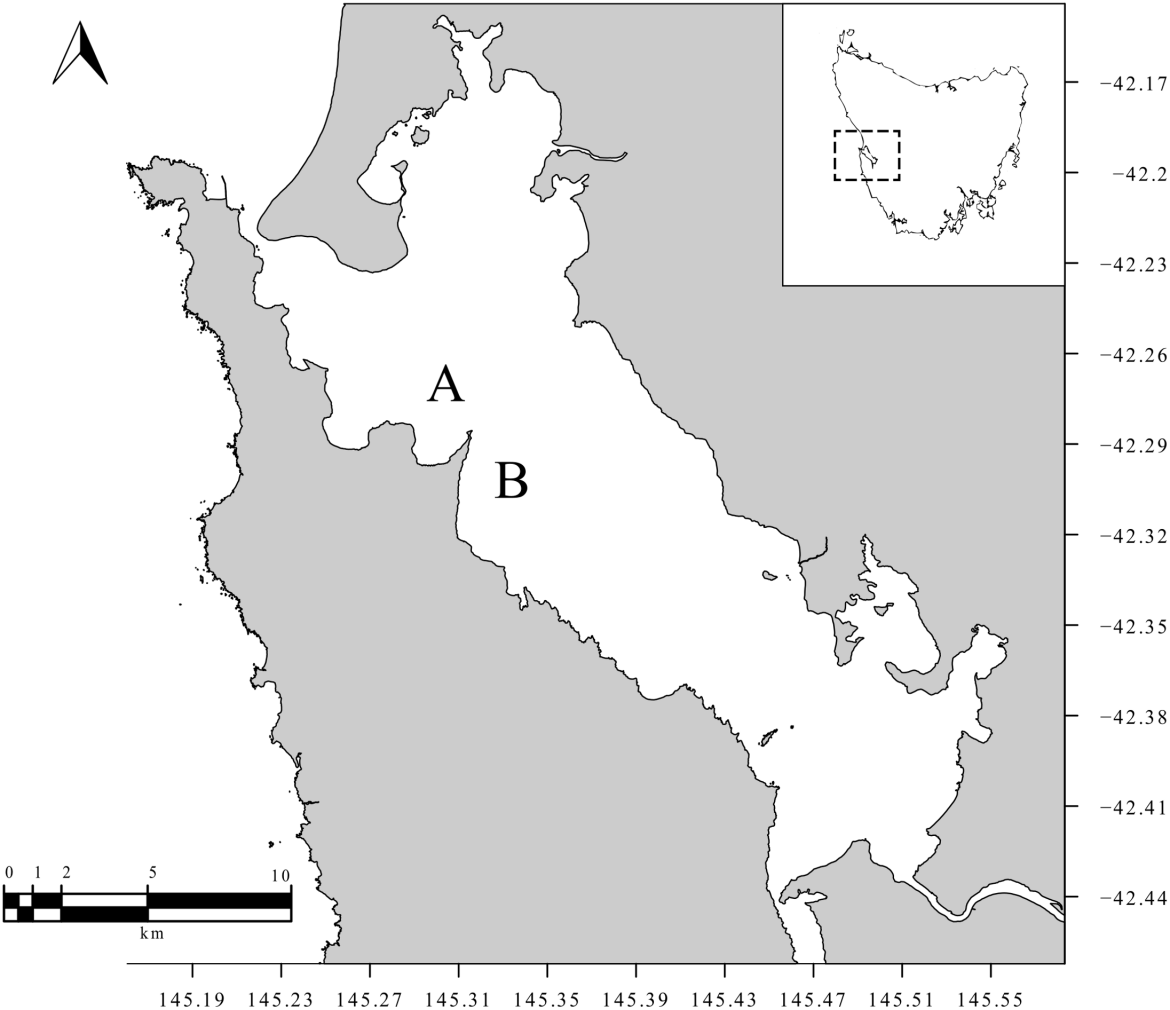
Map of sample sites in Macquarie Harbour (MH). This map shows the location of the two sampling sites used in this study to collect water at depth from Macquarie Harbour (MH), including Table Head (A) and Liberty Point (B). The inset map shows the location of MH on west coast of Tasmania.

To increase the probability of achieving a positive result through detecting *Z*. *maugeana* eDNA in the environment, water samples were collected at two localities within MH where *Z*. *maugeana* was known to be relatively abundant, Table Head (A) and Liberty Point (B) (Fig 1) [27]. *Zearaja maugeana* is a demersal species with a preference for the 10 to 15 m depth range within MH [27]. As a consequence, two 10 L replicate water samples were collected from 10–15 m depths at each site using a Niskin bottle. Each sample was thoroughly shaken to ensure that any eDNA collected was mixed evenly within the bottle, and then separate 1 L, 2 L and 4 L sub-samples were taken from each replicate and stored in plastic bottles. Field sample bottles were stored in the dark in an insulated container containing crushed ice for cooling. To minimize the decay of the eDNA, filtration of the samples commenced within an hour of sample collection but was not competed for all sub-samples until 5 hours after collection.

To determine the decay rate of *Z*. *maugeana* eDNA, and define the detection limits of the assay, water samples were collected from two 1200 L onshore holding tanks that were set up to hold skate individuals for a physiological study. Water for these tanks was pumped from a depth of about 10 m in MH and transported to the holding tanks in preparation for receiving skate specimens. Each tank was sealed and maintained as a closed water system that was monitored for temperature, with a pump placed in each tank to ensure the water was well mixed. With water samples being collected from the closed water system in which *Z*. *maugeana* was known to have been present, these water samples represent the positive controls for the eDNA study.

With evidence of decreasing levels of dissolved oxygen (DO) occurring in MH [29–31], the present study utilized the available physiological experimental design to determine whether DO levels influenced the decay rate of *Z*. *maugeana* eDNA. One tank was maintained at the ambient DO level (55% saturation) within the preferred depth (10–15 m) of *Z*. *maugeana* while the second tank was maintained at a lower DO level (20% saturation). For the physiological study nine *Z*. *maugeana* were captured by gillnet and transported into the two holding tanks, with five individuals (combined biomass of 9431 g) placed into the 55% DO saturation tank and four individuals (combined biomass of 5316 g) placed in the 20% DO saturation tank. After a 48-hour period, the *Z*. *maugeana* individuals were removed from the holding tanks and a 4 L water sample was collected from each tank. Each sample was stored in a plastic container and held at 4°C away from direct sunlight. A 250 ml sub-sample was taken from each 4 L sample container at the start of the decay experiment and then every 24 hours thereafter for three days. Since the eDNA concentrations were expected to be higher than those collected in the field, smaller sub-samples were expected to yield detectable quantities of DNA.

### Sample processing

Following recent work [32] conducted on marine fish eDNA, all water samples were vacuum-filtered using a filtration system that included sterile, disposable 250 ml Nalgene^™^ Analytical Test Filter Funnels (Thermo Scientific) with removable 0.45 μm pore size filters, a vacuum-pump and a collection flask or drum. The eDNA suspended in the water samples was trapped onto the filter. Gloves were worn during filtration and changed in between each sample. To prevent the filters from blocking, only 1 L was filtered per filter paper. After filtration, the filter funnel was discarded and each filter paper containing DNA was removed from the filter cups using sterilized forceps, gently rolled up and placed into a 2 ml cryo-vial. The cryo-vials were stored in liquid nitrogen and transported to an -18°C freezer in the laboratory for further analysis.

A PowerWater DNA Isolation kit (MOBIO laboratories, Qiagen, https://mobio.com/) was used to extract the eDNA by bead-beating action from the filter [4,33]. For samples comprised of more than 1 filter paper (2 L and 4 L environmental samples), the final volumes of all separately extracted samples were combined by ethanol and sodium acetate precipitation to yield a single concentrated eDNA sample in an equivalent final volume (100 μl) as is standard for the kit. The eDNA was extracted from the samples in a dedicated room free from PCR products and extractions were stored at -20°C in a laboratory isolated from the main laboratory.

### Primer design

To ensure the highest sensitivity of detecting *Z*. *maugeana* eDNA in the collected water samples, this study used qPCR to run the assay [4,34]. To design a species-specific qPCR assay for detecting *Z*. *maugeana* in all extracted eDNA samples, this study used mitochondrial primer pairs from the gene region nicotine adenine dinucleotide dehydrogenase subunit 4 (NADH4). This mitochondrial region had previously proven successful in amplifying and sequencing extracted *Z*. *maugeana* DNA for the purpose of a population genetic study of the species (Weltz et al., unpublished data). Additionally, the targeted mitochondrial region has also shown almost no variation in haplotypes of *Z*. *maugeana* in MH, thereby ensuring amplification of any *Z*. *maugeana* eDNA attained from MH (Genbank, accession number KX505075) (Weltz et al., unpublished data).

**Fig 2:**
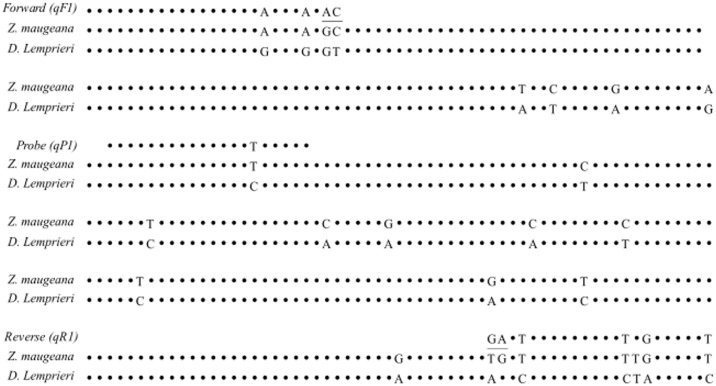
Example of species-specific primer and probe design, using mitochondrial sequence (NADH4 region) aligned between two species. The mitochondrial reference sequence for the Maugean skate (*Z*. *maugeana*) is aligned with the same mitochondrial region occurring in the Thornback skate (*D*. *lemprieri*), with the penultimate bases (underlined) indicating the differences in bp between the two skate sequences. These differences were used to design the selected forward (qF1) and reverse (qR1) primers and a matching probe (qP1) that is specific to *Z*. *maugeana* (product size = 331 bp).

Species-specific MGB (minor groove binder) Taqman^®^ probes were designed inside the NADH4 primer pairs to increase specificity of the qPCR assay. Probes for NADH4 were designed manually using Geneious version 7.5.1 (http://www.geneious.com, [35]. Regions for anchoring the primer and probe sequences were selected by aligning *Z*. *maugeana* sequences with the only known other rajid species present in the Harbour, Thornback skate (*Dipturus lemprieri)*, and choosing conserved areas within *Z*. *maugeana* that differed from *D*. *lemprieri* (Fig 2).

By ensuring that designed primer-probe combinations did not amplify the only other skate occurring in the area, the assay was regarded as specific to the target species [24,36–38]. To further increase the specificity of the assay, the 3’ penultimate base of the primers can be changed to only bind to the target sequence, thereby reducing the risk of mis-priming and mis-amplifying other non-target species [39]. In this study, the 3’ penultimate base of the NADH4 forward and reverse primers was modified to match that of *Z*. *maugeana* and avoid priming with *D*. *lemprieri* (Fig 2).

Specificity for selected primer-probe combinations for NADH4 was further confirmed by testing the qPCR assays, against DNA extracted from other fish species caught in MH, including the rock ling (*Genypterus tigerinus*), greenback flounder (*Rhombosolea tapirina*), elephant fish (*Callorhynchus milii*) and the spiny dogfish (*Squalus acanthias*). The best NADH4 primer-probe pair was selected based on its reaction efficiency in the qPCR assay and specificity to the target species, ensuring the most sensitive assay for *Z*. *maugeana* (Fig 2) [33].

### Real-time PCR (qPCR) conditions

To determine the most sensitive primer-probe combination, the assay was further optimized by varying final primer concentrations between 0.4 μM, 0.8 μM and 1.2 μM per reaction and final probe concentrations between 0.1 μM, 0.2 μM and 0.3 μM of probe. The best combination included primer and probe concentrations of 0.8 μM and 0.2 μM respectively. To provide a standard against which the cycling threshold (CT) values could be produced and final eDNA concentrations could be calculated, a robust positive qPCR standard was optimized from PCR product [40]. The standard consisted of 5 concentrations of 10-fold dilutions spanning four orders of magnitude ranging from 0.00001 ng/μl to 0.1 ng/μl to ensure coverage of possible minimum and maximum eDNA concentration yields of the qPCR runs. All standards maintained high reaction efficiency (1.8)—the efficiency of each cycle to double the amplification product—and an R^2^ > 0.998 to ensure optimal functioning of the qPCR runs. All qPCR reactions were set up using Rotor-Gene 4000 CAS-12000-N (Qiagen, Sydney, Australia) Robotics to ensure consistency in setting up the qPCR runs. The qPCR cycle was optimized to consist of an initial denature step of 95°C for 5 min, followed by a standard 2 step cycle repeated 45 times. The 2 step cycle consisted of a denaturation step of 95°C for 20 sec and a one temperature annealing and extension step of 60°C for 50 sec. All reactions were screened for 6-carboxyfluorescein (FAM) signals with a Rotor-Gene 6000 RG 3000 machine and software (Qiagen, Sydney, Australia) and at the end of each extension step a FAM reading was acquired.

Each qPCR run was comprised of 10 standards, 6 qPCR replicates for each water eDNA sample and 2 qPCR replicates for the negatives. Template qPCR replicates were used to investigate whether there was variation in eDNA yield as a consequence of pipetting eDNA into the reaction. Reactions of final volume 10 μl for the decay experiment were comprised of 0.8 μM primers, 0.2 μM probe, 5 μl of Sensifast2xProbeNo-ROX kit (Bioline, Alexandria, NSA, Australia), 2 μl template and 1.2 μl distilled water. The 10 μl reactions for environmental samples were comprised of 0.8 μM primers, 0.2 μM probe, 5ul of Sensifast2xProbeNo-ROX kit and 3.2 μl of eDNA template. The template sample was increased to 3.2 μl to include as much template as possible in each reaction. To confirm qPCR product identity as *Z*. *maugeana* eDNA, three of the six qPCR replicates with the highest copy number per μl were selected and their DNA was sequenced and alignment to the *Z*. *maugeana* target sequences by the Australian Genome Research Facility (AGRF), Melbourne, Victoria, Australia.

### Contamination control

To minimize contamination of sampling bottles during sample collection by cross-contamination of previously collected water, each bottle was thoroughly sterilized with a detergent (1% Deacon 90) and rinsed with milli-q distilled water prior to each use. To further prevent any contamination by previous samples and to ensure the sample collected only consisted of that sample, bottles were rinsed once with the water being sampled before collecting the sample. Water samples were immediately sealed, ensuring no cross-contamination between samples. Although recently published literature [41] suggests the use of negative controls, given the strict contamination protocol used and the fact that the assay is designed to be species-specific to *Z*. *maugeana*, negatives were not collected in the field.

To prevent contamination of water samples during filtration by DNA existing in the environment or by cross-contamination between samples, this study used disposable filter funnels. A new filter funnel was used to filter each 1 L of water. To further minimize cross-contamination between samples, flame-sterilized stainless steel tweezers were used to remove the filter paper from the disposable filter funnels after each filtration. Similarly, latex gloves were changed between filtration of each new sample.

To prevent contamination during eDNA extractions and laboratory procedures (PCR and qPCR) by any other DNA previously extracted from tissue in the laboratory, a strict laboratory protocol was specifically designed for this study. Separate laboratory rooms located on different levels of the building were used for PCR and qPCR runs, with no overlap between DNA extraction areas. Equipping each room with its own laboratory equipment and personal equipment, including tips, pipettes, gloves and lab coat, any exchange was minimized between the two rooms. Co-workers working in both rooms were made aware of the study and its contamination risks and were prohibited from coming in contact with the eDNA experiments. To minimize human error and contamination while setting up PCR and qPCR reactions, a Rotor-Gene 4000 CAS-12000 robot (Corbett Robotics, Brisbane, Queensland, Australia) was used and was sterilized using UV before each run.

### Data analysis

For each qPCR run, the optimal cycling threshold was determined by autocorrecting to the best fit based on the designed standards. The threshold cycle (Ct), the efficiency of the standard (R^2^) and the reaction efficiency of each reaction (RE) were determined for each qPCR run by conducting post-run quantitative analyses using the Rotor-Gene 6000 software [40]. The threshold cycle defines the number of cycles it takes to detect a fluorescent signal for the target species, as opposed to common background fluorescent noise. Representing the inverse of the amount of nucleic acid present in the sample, Ct values below 29 cycles are defined as having large amounts of DNA copies and Ct values above 38 cycles as having minimal amounts of target DNA copies present in the sample [40]. The R^2^ and RE provide information on the efficiency of the qPCR standards and the efficiency of each reaction run of qPCR [40]. For this study, eDNA from *Z*. *maugeana* was expected to be in diluted quantities in MH water samples thus the maximum Ct value for this study was set to 35 cycles, excluding any reactions amplifying later than this cycle [40]. Post run modifications included no slope correction, normalisation from cycle 1 and dynamic tube normalization [40].

Final *Z*. *maugeana* eDNA concentrations in copies of target eDNA per μl per reaction were calculated from the standards following the methods of Thomsen et al., 2012 [4]. This was determined by comparing template sample amplification against the *Z*. *maugeana* DNA concentration standard curve, described by the Rotor-Gene 6000 software. The *Z*. *maugeana* eDNA concentration for the first standard (0.1 ng/μl) was determined using the following equation [4]:

$$C=\frac{X\left(6.0221*10^{23}\right)}{\left(N*660\right)*10^{9}}$$

Where C = number of copies/μl, X = amount of amplicon (μl), N = length of double stranded NA amplicon (330 base pairs for F1, R1), 660 = average mass of 1bp of ds DNA, 6.0221x10^23^ = Avogadro’s number.

The first standard was used to infer the final *Z*. *maugeana* eDNA concentration for all remaining standards and template reactions. To investigate the relationship between sample volume and eDNA concentration, the *Z*. *maugeana* eDNA concentrations from different water volumes were compared by standardizing sample volumes to amount of eDNA concentration per 250 ml of water.

#### Exponential decay model

To identify the time after excretion (in days) at which *Z*. *maugeana* eDNA was no longer detectable using this assay, a method that was previously employed in an eDNA study on marine fish eDNA was used [4]. Different models were fitted to the data to determine the shape that best described DNA decay in R [42] (S1 File). The model that best fit the data was a generalized linear model (GLM) with an identity link on the log-transformed data, which results in an exponential decay curve:

$$N\left(t\right)=N_{0}e^{-}$$

Where N (*t*) is the concentration at time *t*, N_0_ is the eDNA concentration at time = 0, and β is the decay rate.

The detection threshold was defined using the concentration of the lowest qPCR standard (0.0001ng/μl) at which reliable results were achieved for qPCR runs. The detection threshold was used to determine the lowest eDNA concentration (eDNA copies/μl) at which the qPCR assay used in this study could detect *Z*. *maugeana* eDNA. This, in combination with solving for time (t) in the exponential decay model equation, provided the time (days) at which the eDNA concentration of *Z*. *maugeana* fell below the detection threshold of the assay.

To examine the potential impact of varying DO levels in MH on the decay rate of *Z*. *maugeana* eDNA samples collected from the wild, both models for ambient DO levels (55% saturation) and lower DO levels (20% saturation) were applied to the field data. The starting concentration for each simulation was chosen as the maximum eDNA concentration attained at either site, as this represents the highest chance of detecting *Z*. *maugeana* eDNA using this assay.

## Results

### Field samples

Environmental DNA from *Z*. *maugeana* was successfully amplified from all of the field collection samples, with the exception of one of the 4 L replicates from site A, which was compromised during the extraction process and therefore excluded. All qPCR reactions showed target amplification below 31 Ct and negative controls exhibited no amplification. The efficiency of each qPCR was high, with R^2^ values of 0.95 and 0.85 for the assays conducted for sites A and B, respectively. Reaction efficiencies (RE) were different between the sample replicates within sites A and B, ranging on average from 44%-100% efficiency of doubling of the amplicon product. To confirm that qPCR results were in fact of the target species, qPCR products of the assay were sequenced and aligned with the known target *Z*. *maugeana* DNA sequence [43,44]. This alignment revealed a 100% match between the PCR product and the *Z*. *maugeana* DNA target sequence.

The eDNA concentrations acquired varied between sites and between replicates within each site (Table 1). As expected, an increase in the volume of water filtered increased the quantity of *Z*. *maugeana* eDNA acquired; with 4L samples having the highest concentrations of target eDNA in Table 1. However, when standardized for sample volume, eDNA concentrations were less variable within a replicate.

**Table 1:** Average eDNA concentration (copies per μl) extracted from water samples collected in MH. Sample sites Table Head (site A) and Liberty Point (site B) along with sample replicates, filter volumes (L), mean eDNA concentrations (copies per μl) of multiple qPCR runs of a single sample volume and the standard error (Std error) of that mean. Additionally, both mean and standard error have been standardized to samples of 250ml.

Site	Sample replicate	Filter Volume	Mean eDNA concentration (x10^3) ± Std error	Standardized to 250ml eDNA concentration (x10^3) ± Std error
A	1	1	225.68 ± 10.94	56.42 ± 2.74
A	1	2	475.36 ± 29.92	59.42 ± 3.74
A	2	1	5.10 ± 0.91	1.27 ± 0.21
A	2	2	8.86 ± 0.88	1.11 ± 0.11
A	2	4	13.64 ± 0.64	0.85 ± 0.04
B	1	1	51.07 ± 6.82	12.77 ± 1.70
B	1	2	363.35 ± 81.88	45.42 ± 10.24
B	1	4	562.28 ± 28.29	35.14 ± 1.94
B	2	1	87.31 ± 7.01	21.83 ± 1.75
B	2	2	441.34 ± 34.02	55.17 ± 4.66
B	2	4	1717.25 ± 218.35	107.33 ± 14.95

### Decay experiment

*Zearaja maugeana* eDNA was successfully amplified from all subsamples (250ml), with Ct scores for tanks with DO levels of 55% saturation and 20% saturation ranging from 15.52–27.26 and 14.52–32.13 respectively. The R^2^ values were above 0.99, there was no amplification in the negative controls and the qPCR products showed 100% match to *Z*. *maugeana* target sequence.

**Fig 3:**
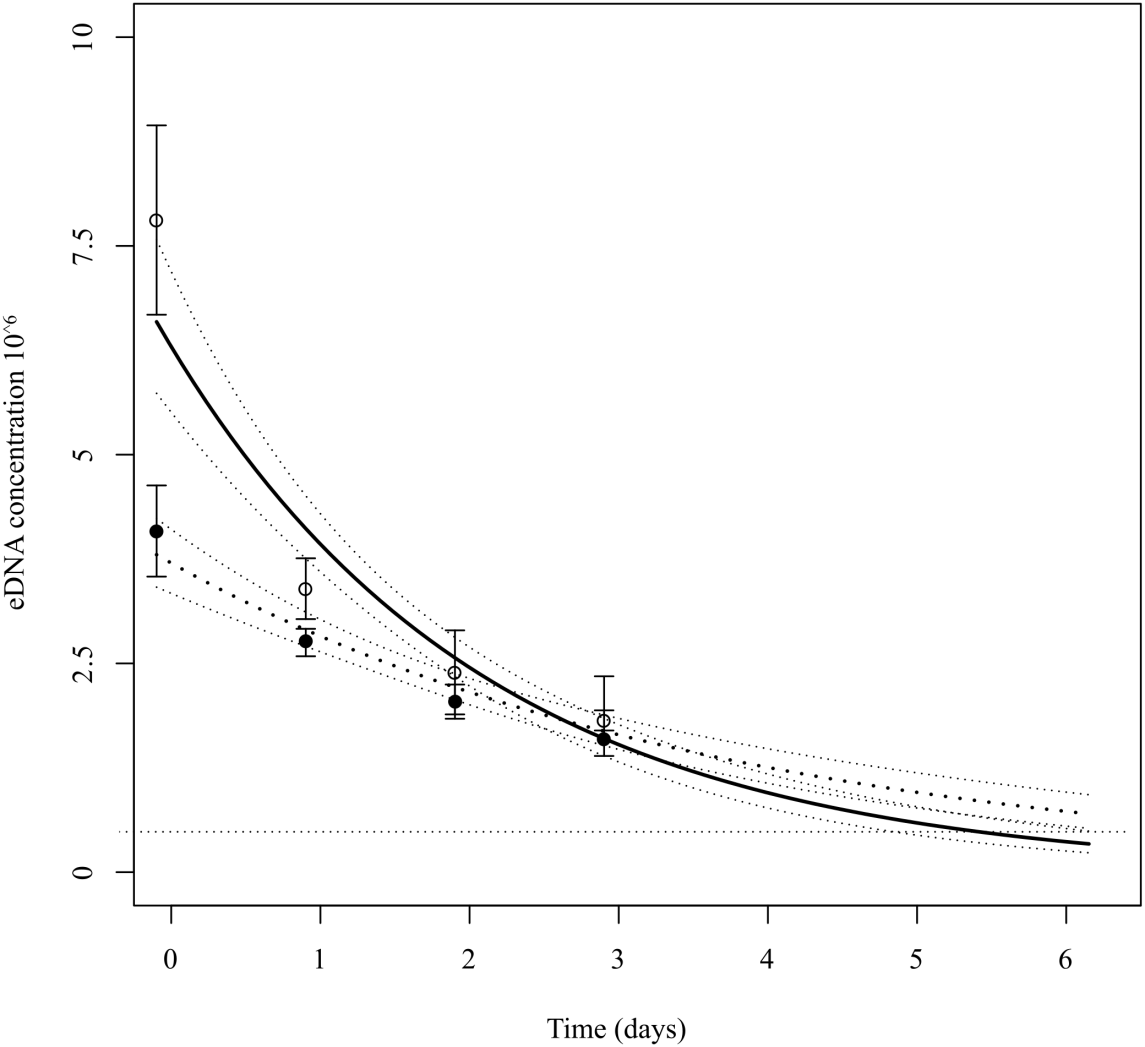
Exponential decay rate of *Z*. *maugeana* eDNA concentrations (copies per μl) over time. For both DO treatments, including 55% saturation DO (---) and 20% saturation DO (—), *Z*. *maugeana* eDNA was still detected after 3 days using the assay and primer and probe pairs designed in this study.

The two tanks showed noticeably different initial eDNA concentrations, with the tank with a higher total biomass of *Z*. *maugeana* showing a higher initial eDNA concentration than the tank with lower biomass (Fig 3). Regardless of the initial concentration, fitting the exponential decay model to both tank treatments of ambient DO levels (55% saturation) (F(1,22) = 159.86, p<0.001, adjR^2^ = 0.86) and lower DO levels (20% saturation) (F(1,22) = 84.29, p<0.001, adjR^2^ = 0.78), suggested that both DO treatments showed a significant decrease in eDNA concentration over time, with *Z*. *maugeana* eDNA still being detectable after 3 days for both experiments (Fig 3).

Despite not observing data beyond 3 days, assuming the rate remains constant, the model would predict that the eDNA concentration drops below the detection threshold of the assay after 5 days at ambient DO (55% saturation) and 7 days at lower DO (20% saturation) (Fig 3). Furthermore, the rate of decay (β) differed slightly between the two tanks, with the eDNA from the tank with ambient DO levels (55% saturation) (β = -0.020) decaying at a faster rate initially than the eDNA from the tank with lower DO levels (20% saturation) (β = -0.011) (Fig 3).

Applying the decay model determined for ambient DO levels (55% saturation) to the eDNA data collected a 250ml field sample, suggested that the earliest point at which the eDNA concentration drops below the detection limit of the assay was 4 and 16 hours for site A and B respectively. Applying the decay model determined for lower DO levels (20% saturation) to the same eDNA field data, showed that the earliest point at which the eDNA concentration drops below the detection limit of the assay was 12 and 60 hours for site A and B respectively.

## Discussion

The eDNA of *Z*. *maugeana* was successfully extracted and amplified from water samples collected from MH. The successful amplification confirms that the eDNA assay designed in this study can be used to detect the presence of this rare elasmobranch in the wild. This application supports previous studies applying eDNA techniques to determine the presence of rare elasmobranch species in their natural habitat, including whale sharks [8] and the largetooth sawfish [24]. These studies re-enforce the potential for eDNA as an alternate approach to traditional methods for surveying threatened elasmobranch species in the marine environment.

In addition to determining presence, eDNA has also been suggested to be useful in assessing the abundance of the target species in the field [32,45]. Previous studies have indicated that higher densities result in an increase in the amount of eDNA produced in the environment [46]. In this study, a higher initial eDNA concentration was observed for the tank with a higher total biomass of *Z*. *maugeana* during the decay experiment.

Although the assay designed in this study is able to quantify the amount of eDNA concentration detected in the water samples, it is unlikely that this can be used to estimate absolute abundance of *Z*. *maugeana* in MH. This is attributed to the fact that the eDNA detected in the water samples could have originated from one or multiple individuals. Moreover, with the age of the eDNA detected in the water samples being unknown, it is uncertain as to how much the eDNA has degraded in the environment prior to being sampled. Without taking the age of the eDNA into consideration, this could lead to under-estimation of abundance of *Z*. *maugeana* in the area at the time of sampling. Finally, using this assay to determine absolute abundance estimates for *Z*. *maugeana* would be assuming that all individuals shed an equal amount of eDNA at an equal rate. Although the amount and rate of eDNA being shed by marine species has not yet been compared between multiple individuals, it is likely that this will differ between individuals based on physiological characteristics that may influence eDNA shedding, including size, health or possibly even reproductive status.

However, a recent field study on the Japanese jack mackerel (*Trachurus japonicus*), demonstrated, that eDNA concentrations acquired from water samples collected at the surface were positively correlated with relative biomass of the target species at depth in the area at the time of sampling [47]. Given that MH is a unique system with a stable, deep marine water layer in which *Z*. *maugeana* has been shown to be resident year round, this may be the ideal environment to test whether variability in eDNA concentrations could provide a proxy for variability in the relative abundance of *Z*. *maugeana* between sites and different habitats.

Environmental DNA has recently been used to investigate more specific population genetic characteristics of rare elasmobranchs in the wild, based on comparing mitochondrial eDNA haplotype frequencies detected in seawater to those acquired from tissue samples [8]. However, since there is almost no variation in mitochondrial haplotypes of *Z*. *maugeana* sampled in MH (Weltz et al., unpublished data), this approach is unlikely to be informative. Nevertheless, the question as to whether *Z*. *maugeana* still occurs in BH remains, and if present there is also the question of the genetic relationship between the populations. Although originally described from BH, no individuals have been recorded from that locality since 1992 despite a number of faunal surveys conducted in the area [26]. This has led to the suggestion that *Z*. *maugeana* is either no longer extant in BH or that the population is extremely small and hence vulnerable [26]. With traditional methods, including diving and fishing surveys, failing to confirm the presence of *Z*. *maugeana* in BH, this study recommends using eDNA as a feasible alternative survey method to try and detect *Z*. *maugeana* in this area. However, when interpreting eDNA results for especially rare species it is important to consider that while a positive result can confirm the presence of the target species, a negative result does not necessary confirm that the species does not occur in the sampling area. A negative eDNA result can be due to the target species not being present in the area of sampling, at the time of sampling, or that the eDNA is in such low concentrations as to be below the detection threshold of the assay [36].

Thus to optimize the application of eDNA it is important to understand how factors, such as sample volumes and the decay of eDNA, can influence eDNA concentrations. For instance, the present study established that concentrations of *Z*. *maugeana* eDNA collected in the field have the potential to decay beyond the detection limit within as little as 4 hours after sampling. This is supported by previous work on elasmobranchs, in which the eDNA of a skate species, *Raja typus*, was shown to decay beyond detection within days of sampling [8]. The time it takes for eDNA to decay beyond detectable limits is not only dependent on the starting concentrations of eDNA within the sample but also influenced by environmental conditions, including the dissolved oxygen concentration of the sample. Although *Z*. *maugeana* eDNA was amplified from as little as 1 L of water collected in the field, it is recommended that in locations where the presence of the species is uncertain, or likely to be in very low abundances, that larger volumes are sampled and processed as soon as practicable. This strategy should increase the probability of establishing valid conclusions [4,20]. However, there is a trade off between the time taken to filter the sample and the volume of water in the sample. Thus, a system capable of filtering multiple samples simultaneously would be highly recommended. It is also recommended that processing times be kept under 3 hours, since decay modelling suggested that some samples would have fallen below the detection limit of the assay within 4 hours of sampling. The fact that processing times ranged up to five hours in this study, may explain some of the observed variability in eDNA concentration between replicate samples.

Furthermore, although it has been suggested that negative controls should be included in future eDNA work, the results of this study suggest no contamination based on the variability in the concentration of eDNA acquired between and within samples and sites in MH [41]. Moreover, the fact that eDNA concentrations were, as expected, far lower in the field compared to concentrations in the experimental setting, instills confidence in the results.

Finally, this study revealed considerable variation in eDNA concentrations between replicates at individual sites as well as between sampling sites. It is, therefore, recommended that multiple samples be taken from within the study area or habitats of interest. For rare species, such as *Z*. *maugeana*, a greater number of samples will provide increased confidence in being able to confirm the presence of the target species or at least the possibility that it is not present in the area. The successful application of the eDNA technique will likely dictate whether BH should be included in future management plans for *Z*. *maugeana* as well as confirming the significance and vulnerability of the population in MH in terms of the future viability of this unique, endangered species.

## Supporting information

S1 Data Excel spreadsheet containing the raw Real-time PCR (qPCR) data acquired from water samples collected in Macquarie Harbour. The data consists of the eDNA concentration (copies eDNA molecules per micro-liter) acquired from water samples collected in the field at depth at sample sites Table Head (THD) and Liberty Point (LP). Average eDNA concentrations (copies eDNA molecules per micro-liter) and their corresponding standard deviations were determined for sample volumes of 1 L, 2 L and 4 L, as well as standardizing these to 250ml across all samples.
(XLSX)

S2 Data Excel spreadsheet containing the raw Real-time PCR (qPCR) data acquired from water samples collected in the exponential decay tank experiments. The data consists of the eDNA concentration (copies eDNA molecules per micro-liter) acquired from water samples taken from two experimental tanks in which *Z*. *maugeana* were kept for 48 hours and the removed. Water samples (250ml) were collected from both tanks over a time period of 72 hours, allowing for any possible eDNA decay to occur within this time-frame.
(XLSX)

S1 File R program file for running the exponential decay model in R. The file contains the R code used in this study to run the exponential decay model to determine the decay rate of eDNA concentrations over a time period of 72 hours using the data from S1 Data.
(R) Click here for additional data file.
